# Genome sequences of the first Autographiviridae phages infecting marine Roseobacter

**DOI:** 10.1099/mgen.0.001240

**Published:** 2024-04-17

**Authors:** Sen Du, Ying Wu, Hanqi Ying, Zuqing Wu, Mingyu Yang, Feng Chen, Jiabing Shao, He Liu, Zefeng Zhang, Yanlin Zhao

**Affiliations:** 1 College of Juncao Science and Ecology, Fujian Agriculture and Forestry University, Fuzhou, PR China; 2 Institute of Marine and Environmental Technology, University of Maryland Center for Environmental Science, Baltimore, Maryland, USA; 3 Key Laboratory of Marine Biotechnology of Fujian Province, Institute of Oceanology, Fujian Agriculture and Forestry University, Fuzhou, PR China

**Keywords:** *Autographiviridae *phages, *Roseobacter*, comparative genomics, distribution patterns, phylogenomic analysis

## Abstract

The ubiquitous and abundant marine phages play critical roles in shaping the composition and function of bacterial communities, impacting biogeochemical cycling in marine ecosystems. *Autographiviridae* is among the most abundant and ubiquitous phage families in the ocean. However, studies on the diversity and ecology of *Autographiviridae* phages in marine environments are restricted to isolates that infect SAR11 bacteria and cyanobacteria. In this study, ten new roseophages that infect marine *Roseobacter* strains were isolated from coastal waters. These new roseophages have a genome size ranging from 38 917 to 42 634 bp and G+C content of 44.6–50 %. Comparative genomics showed that they are similar to known *Autographiviridae* phages regarding gene content and architecture, thus representing the first *Autographiviridae* roseophages. Phylogenomic analysis based on concatenated conserved genes showed that the ten roseophages form three distinct subgroups within the *Autographiviridae*, and sequence analysis revealed that they belong to eight new genera. Finally, viromic read-mapping showed that these new *Autographiviridae* phages are widely distributed in global oceans, mostly inhabiting polar and estuarine locations. This study has expanded the current understanding of the genomic diversity, evolution and ecology of *Autographiviridae* phages and roseophages. We suggest that *Autographiviridae* phages play important roles in the mortality and community structure of roseobacters, and have broad ecological applications.

**Impact Statement**

We report here the first study of *Autographiviridae* phages infecting marine *Roseobacter* strains. By characterizing these novel roseophages, we have expanded the knowledge of marine *Autographiviridae* phages beyond previously studied *Autographiviridae* cyanophages and pelagiphages. Comparative genomic and phylogenomic analyses of these roseophages have led to the identification of three new subgroups and eight new genera within the family *Autographiviridae*. Furthermore, a viromic read-mapping analysis indicated that these *Autographiviridae* roseophages are predominantly distributed in polar and estuarine environments. This work contributes significantly to the existing knowledge of the genomic diversity and ecology of marine *Autographiviridae* phages.

## Introduction

Bacteriophages (hereafter phages) are the most numerically abundant and genetically diverse biological life forms on earth and in the oceans [1,2]. They dominate marine viral communities and play essential roles in modulating mortality, community structure and evolution of microbial communities [2]. Phages impact the biogeochemical cycling of carbon and nutrients by lysing bacteria, releasing cellular carbon and nutrients, and altering the metabolism of infected hosts [3,9].

The rapidly growing quantity of viromic data has led to increasing insights into the community composition of marine viral communities and the identification of many abundant and important phage groups [10,15]. Among diverse phage groups, the well-known family *Autographiviridae* (belonging to the realm *Duplodnaviria*, kingdom *Heunggongvirae*, phylum *Uroviricota*, class *Caudoviricetes*) is one of the most ubiquitous phage families in the ocean [11,16,18]. *Autographiviridae* phages are characterized by the possession of a single RNA polymerase (RNAP) gene that transcribes phage class II genes [19]. Before being assigned as a family*, Autographivirinae* was previously known as the ‘T7 supergroup’, and it was then assigned to the subfamily *Autographivirinae* under the family *Podoviridae* [20,21]. The genomes of more than 60 marine *Autographiviridae* isolates, most of which were isolated from *Cyanobacteria* (*Synechococcus* and *Prochlorococcus*) and SAR11 bacteria (order *Pelagibacterales*), are available in the non-redundant database (nr) of the NCBI (National Center for Biotechnology Information) to date [18,22,29]. Current studies on the diversity, evolutionary relationships and ecology of marine *Autographiviridae* phages are restricted to cyanophages and pelagiphages (infecting marine unicellular cyanobacteria and SAR11 bacteria, respectively) [18,23,29]. Some *Autographiviridae* cyanophages and pelagiphages have the potential to lysogenize their hosts [28]. In addition, *Autographiviridae* cyanophages have been reported to reprogram host metabolism by causing differential expression of host genes and expressing phage-encoded auxiliary metabolic genes (AMGs) during infection [27]. Despite this progress on marine *Autographiviridae* phages, studies on *Autographiviridae* phages infecting other marine bacteria are lacking.

The *Roseobacter* cluster (family *Roseobacteraceae*) is one of the most ecologically important bacterial groups in marine environments [30,31]. Roseobacters are abundant and dominant in coastal and polar environments. They are metabolically versatile and can utilize diverse organic compounds, thus playing important roles in global carbon and sulphur cycling [30,34]. Diverse phages infecting *Roseobacter* (termed roseophages) have been isolated and studied [35,40]. However, none of them belongs to the family *Autographiviridae*.

In this study, using strains of several dominant *Roseobacter* lineages as hosts, we isolated and genomically characterized the first *Autographiviridae* roseophages to gain a greater understanding of marine *Autographiviridae* phages and roseophages. Comparative genomic and phylogenomic analyses revealed that they display conserved genome architecture and evolutionary relationships with other known *Autographiviridae* phages and represent three new subgroups and eight new genera within the *Autographiviridae*. Viromic read-mapping analysis showed that these new *Autographiviridae* roseophages were mainly distributed in polar and estuarine environments.

## Methods

### Host strains, growth media and growth conditions

By employing the dilution-to-extinction method [41], three *Roseobacter* RCA strains (FZCC0023, FZCC0042, FZCC0040) [38], and a new *Roseobacter* strain FZCC0037 were isolated in May 2017, from the coastal waters of Pingtan Island, China (25° 26′ N 119° 47′ E). *Roseobacter* CHUG strain FZCC0196 was isolated in December 2021, from the coastal waters of Pingtan Island (25° 26′ N 119° 47′ E) using the dilution-to-extinction method [41]. All *Roseobacter* strains were cultured at 23 °C in a sterilized natural seawater-based medium supplemented with 1 mM NH_4_Cl, 100 µM KH_2_PO_4_, 1 µM FeCl_3_ and mixed carbon sources [42]. After the bacterial strains were stained with SYBR Green I (Invitrogen), the bacterial counts were determined using a Guava easyCyte flow cytometer (Merck Millipore).

### Roseophage isolation and purification

Information on the seawater samples collected for roseophage isolation is provided in Table 1. Surface seawater samples were filtered using sterile syringe filters with pore sizes of 0.1 µm and stored in the dark at 4 °C until use. The procedure for isolating phages infecting marine oligotrophic bacteria has been described previously in the literature [26,28, 38]. Briefly, filtered seawater samples were added to exponentially growing host cultures, and cell growth was monitored using a Guava easyCyte flow cytometer. When a decrease in cell density was detected, the release of phage particles was confirmed using epifluorescence microscopy. The phages were purified using the dilution-to-extinction method [26,43]. Whole-genome sequencing and assembly were employed to assess the presence of other viral contamination signatures.

**Table 1.** General characteristics of the ten newly isolated *Autographiviridae* phages infecting marine *Roseobacter*

Phage	Original host	Source water	Depth	Latitude	Longitude	Collection date	Genome size (bp)	No. of ORFs	%G+C
CRP-114	*Roseobacter* FZCC0023	Yantai coast, Bohai Sea	Surface	37° 28′ N	121° 28′ E	April 2021	40 413	50	48.8
CRP-113	*Roseobacter* FZCC0023	Taizhou coast, East China Sea	Surface	28° 18′ N	121° 38′ E	September 2021	39 709	48	44.9
CRP-118	*Roseobacter* FZCC0023	Taizhou coast, East China Sea	Surface	28° 18′ N	121° 38′ E	September 2021	41 088	52	46.4
CRP-171	*Roseobacter* FZCC0023	Ningbo coast, East China Sea	Surface	29° 28′ N	121° 58′ E	September 2021	39 796	48	44.9
CRP-143	*Roseobacter* FZCC0023	Ningbo coast, East China Sea	Surface	29° 28′ N	121° 58′ E	September 2021	42 634	53	44.6
CRP-125	*Roseobacter* FZCC0023	Taizhou coast, East China Sea	Surface	28° 18′ N	121° 38′ E	September 2021	38 917	50	48.7
CRP-403	*Roseobacter* FZCC0037	Pattaya coast, Indian Ocean	Surface	12° 56′ N	100° 53′ E	March 2018	40 567	52	47.0
CRP-227	*Roseobacter* FZCC0040	Yantai coast, Bohai Sea	Surface	37° 28′ N	121° 28′ E	December 2021	39 373	49	48.3
CRP-361	*Roseobacter* FZCC0042	Yantai coast, Bohai Sea	Surface	37° 28′ N	121° 28′ E	December 2021	39 084	47	48.5
CRP-804	*Roseobacter* FZCC0196	Qingdao coast, Yellow Sea	Surface	36° 37′ N	121° 10′ E	September 2021	41 620	52	50.0

### Morphological analysis using transmission electron microscopy

The morphology of the representative roseophages was observed using transmission electron microscopy (TEM). Phage lysates were filtered through 0.1 µm pore-size filters and then concentrated using Amicon Ultra Centrifugal Filters (30 kDa; Merck Millipore). The concentrated phage particles were absorbed onto copper grids in the dark, negatively stained with 2 % (w/v) uranyl acetate for 2 min and air-dried. The samples were observed using a Hitachi transmission electron microscope at an acceleration voltage of 80 kV.

### Phage DNA extraction, genome sequencing and genome assembly

Each phage lysate (~150 ml) was filtered through 0.1 µm filters and concentrated to 1.5 ml by using Amicon Ultra Centrifugal Filters (30 kDa; Millipore). The concentrated lysate was subsequently concentrated by ultracentrifugation (40 000 r.p.m. for 2 h). Phage genomic DNA was extracted using a DNeasy Blood and Tissue Kit (Qiagen) and sequenced on a HiSeq 2500 platform (Illumina) with a paired-end read length of 150 bp. Fastp v0.23.2 was used for quality filtering and trimming (-q 20 -l 50) [44]. The *de novo* genome assembly was performed using MEGAHIT v1.2.9 (--k-list, 21, 29, 39, 59, 79, 99, 119, 141) [45]. The genome sequences of the six roseophages were assembled into circular molecules, suggesting that the genomes are complete. The remaining four genomes contained some gaps after assembly. These gaps were closed through Sanger sequencing of the PCR products covering the gap areas. The position of the primers was determined based on a comparison with other closely related genomes (Table S1, available in the online version of this article).

### Genome annotation and comparative genomic analysis

Prodigal v2.6.3 was used to predict the ORFs of the *Autographiviridae* phage genomes [46]. The translated ORFs were annotated using BLASTp against the NCBI non-redundant and NCBI Refseq (v215) databases (E-value ≤10^−3^, amino acid identity ≥25 % and alignment length ≥50 %). ORFs were searched against the Pfam database using HMMscan to identify conserved Pfam domains (-E 1e-3 -T 50) [47,48]. tRNA was identified using tRNAscan-SE [49]. Representative *Autographiviridae* genomes were compared and visualized using Easyfig v2.2.2 [50].

### Phylogenetic analyses

Maximum-likelihood phylogenetic trees based on DNA polymerase (DNAP) and terminase large subunit (TerL) sequences were reconstructed to evaluate the evolutionary relationships among *Autographiviridae* phages. This analysis consists of DNAP and TerL sequences from selective *Autographiviridae* phages from NCBI RefSeq (v215) and the roseophages isolated in this study. The sequences were aligned using MAFFT v7.310 (--maxiterate 1 000 --localpair) [51] and edited using trimAl v1.4.rev15 (-automated1) [52]. Phylogenetic trees were reconstructed using IQ-TREE v2.2.0.3 [53].

A genome-wide proteomic tree was reconstructed using ViPTree [54], which was calculated by tBLASTx for genome-wide sequence similarities. The intergenomic similarities of isolated roseophages and related *Autographiviridae* cyaophages and pelagiphages were calculated by VIRIDIC [55].

Seven conserved genes (RNAP, DNAP, portal protein, capsid protein, tail tubular protein A, tail tubular protein B, TerL) from marine *Autographiviridae* phages were selected for phylogenomic analysis. The amino acid sequences of the seven core genes were aligned using MAFFT and edited using trimAl. Thereafter, alignment was used to reconstruct a phylogenomic tree using IQ-TREE with 1000 bootstrap replicates. The phylogenetic trees were visualized using the Interactive Tree Of Life (iTOL) v6 [56].

### Viromic read recruitment analysis and statistical analysis

The relative abundance of the *Autographiviridae* roseophages was estimated using a viromic read-mapping analysis. A total of 220 marine viromic datasets, including Global Ocean Viromes 2.0[13], Pearl River estuary virome [57], Mariana Trench virome [58], Eastern Tropical North Pacific virome [59], Delaware Bay and Chesapeake Bay viromes [60], Black Sea virome [61], Red Sea virome [62] and South China Sea DNA virome [63], were used for viromic read-mapping analysis.

Viromic reads were mapped against the non-redundant set of analysed *Autographivirida*e genomes using coverM (-p bwa-mem --min-read-percent-identity 95 --min-read-aligned-length 50 --min-read-aligned-percent 80, https://github.com/wwood/CoverM). The relative abundance of these phages was normalized by RPKM (reads per kilobase per million reads in the viromic dataset). A heatmap of the RPKM of phages was generated using the pheatmap package in R [64]. A linear-regression analysis generated using R was used to evaluate the relationship between environmental parameters and the relative abundance of these phages. Statistical significance was set at *P*<0.05.

## Results and discussion

### Host strains

Five *Roseobacter* strains (FZCC0196, FZCC0023, FZCC0037, FZCC0040 and FZCC0042) were used as hosts for phage isolation. FZCC0023, FZCC0040 and FZCC0042 belong to the *Roseobacter* RCA lineage, and the phylogenetic information of these RCA strains was described in a previous report [38]. FZCC0037 represents a new *Roseobacter* lineage (Fig. S1). FZCC0196 shares 99.62 % 16S rRNA gene sequence similarity with the *Roseobacter* CHUG strain HKCCA1288 [65] and clusters with CHUG strains in the 16S rRNA gene tree (Fig. S1). Thus, it also belongs to the CHUG lineage.

### General characterization of newly isolated phages

**Fig. 1.**
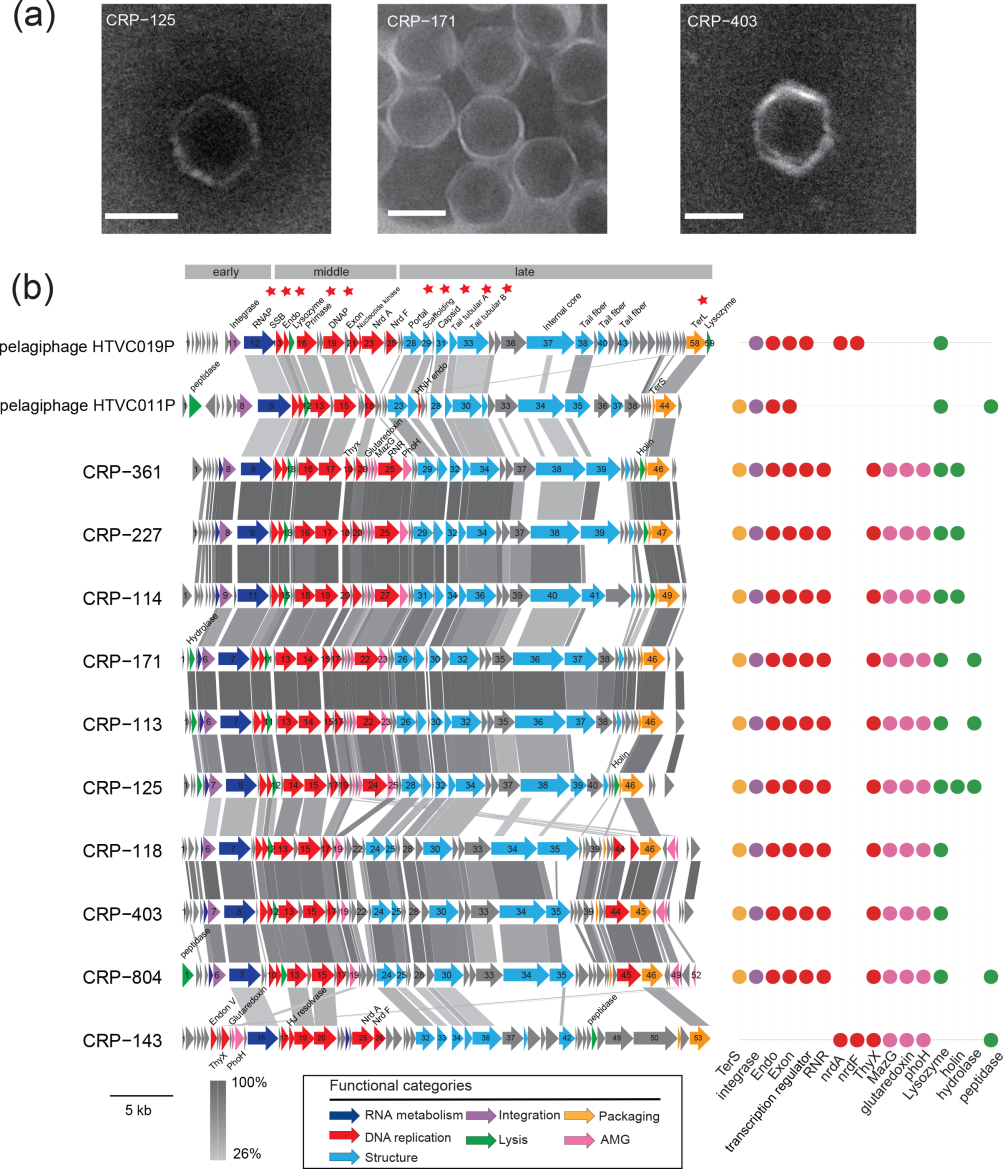
Morphylogy and genome map of *Autographiviridae* roseophages sequenced in this study. (**a**) Transmission electron microscope images of representative roseophages isolated in this study. Bars, 50 nm. (**b**) Genome comparison of the ten *Autographiviridae* roseophages and *Autographiviridae* pelagiphages. Predicted ORFs are represented by arrows and coloured based on their putative functions. The scale colour bar indicates amino acid identities between homologous genes. Conserved essential genes shared by all roseophages are indicated by red asterisks. The distribution of other functional genes is indicated. RNAP, RNA polymerase; SSB, single-stranded DNA binding protein; DNAP, DNA polymerase; Endo, endonuclease; TerS, terminase small subunit; TerL, terminase large subunit; MazG, MazG-like pyrophosphatase gene; RNR, ribonucleotide reductases; PhoH, starvation-inducible protein gene; ThyX, thymidylate synthase; NrdA, ribonucleotide-diphosphate reductase alpha subunit; NrdF, ribonucleotide-diphosphate reductase beta subunit.

**Fig. 2.**
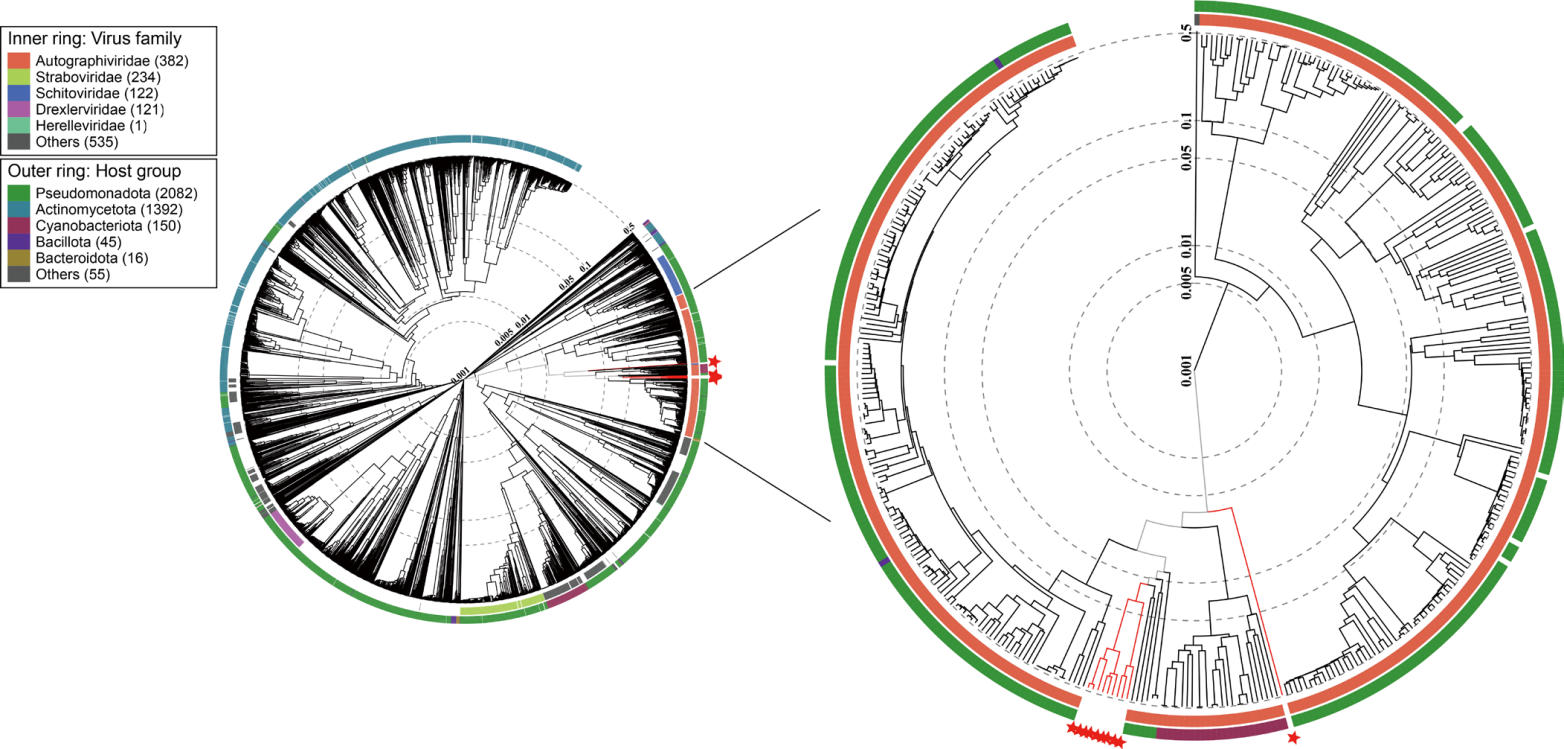
Genome-wide proteomic tree reconstructed using VipTree for the new roseophages isolated in this study and other related known prokaryotic dsDNA phages. The coloured inner and outer rings represent the virus family and host groups. Branches coloured red represent the newly isolated phages in this study.

Here, ten phages infecting *Roseobacter* strains were isolated from coastal waters (Table 1). TEM showed that they have an icosahedral capsid (ca. 60 nm in diameter) and a short tail (Fig. 1a). The genome sizes of these roseophages ranged from 38 917 to 42 634 bp, with G+C content ranging from 44.6 to 50.0 %, similar to the G+C content of their hosts (44.8–54.1 %). These phages were predicted to harbour 47–53 ORFs (Table 1). Genome annotation assigned putative functions to approximately 40 % of the ORFs in each genome, while the remaining ORFs were classified as hypothetical proteins without known functions. The majority of the function-known genes in these roseophages are related to DNA metabolism and replication, gene transcription, phage structure, and DNA packaging. No tRNA was identified in their genomes. Genome comparison revealed that these roseophage genomes exhibited considerable similarities in their gene content and genome architecture (Fig. 1b). They all possessed homologues of essential phage reproduction genes of *Autographiviridae* phages [20], including genes encoding RNAP, DNAP, DNA primase, single-stranded DNA binding protein (SSB), portal protein, tail tube protein, major capsid and TerL (Fig. 1b). This result suggests that they are all evolutionarily related to *Autographiviridae* phages. A genome-wide evolutionary analysis using ViPTree showed that these newly isolated roseophages are located within the known *Autographiviridae* phages (Fig. 2), suggesting they are all new members of the family *Autographiviridae*. This study is the first to report *Autographiviridae* phages infecting marine *Roseobacter* strains.

Similar to other known *Autographiviridae* phages, genes of these ten phages can be classified into three consecutively arranged functional modules: the host takeover module (early genes), the DNA replication and metabolism module (middle genes), and the morphogenesis module (late genes) (Fig. 1b). The early regions of these phages comprise an RNAP gene and several small genes with unknown functions. The RNAP gene is considered as the defining characteristic of the family *Autographiviridae* and has been reported to be responsible for middle gene transcription in *Escherichia coli* phage T7 [19,21]. Additionally, nine of the ten roseophages harbour a tyrosine family integrase gene. Integrase genes catalysing the phage–host genome integration are widespread in marine *Autographiviridae* pelagiphage and cyaophage genomes [18,23,29]. The presence of the integrase gene implies the lysogeny potential of these *Autographiviridae* roseophages.

In the DNA replication and metabolism module, all ten roseophages contain a full set of typical T7-like DNA replication-related genes located downstream of the RNAP gene, including genes encoding SSB, endonuclease, DNA primase/helicase, DNAP and exonuclease, suggesting that they share a conserved DNA replication machinery. Phylogenetic analysis based on the DNAP gene revealed that these new phages clustered with *Autographiviridae* phages and were closely related to *Autographiviridae* cyanophages (Fig. S2). Genes encoding thymidylate synthase (ThyX) were present in all ten roseophage genomes. ThyX is responsible for catalysing the biosynthesis of deoxythymidine monophosphate and is important in DNA replication and repair [66,67]. Genes encoding class I ribonucleotide reductases (RNRs) were identified in the CRP-143 genome, whereas the other nine roseophages contain a class II adenosylcobalamin-dependent RNR gene. RNRs are essential enzymes that catalyse the conversion of nucleotides to deoxynucleotides, therefore playing critical roles in DNA replication and repair [68,70].

In the morphogenesis module, all *Autographiviridae* roseophages possess a set of conserved genes encoding portal protein, scaffolding protein, capsid protein, tail tubular proteins A and B, and TerL. This suggests that they all have a conserved T7-like head–neck–tail module. TerL phylogenic analysis revealed that *Autographiviridae* roseophages are also closely related to *Autographiviridae* cyanophages (Fig. S2). Additionally, several lysis genes were identified within this module.

### Phylogenomic analyses

**Fig. 3.**
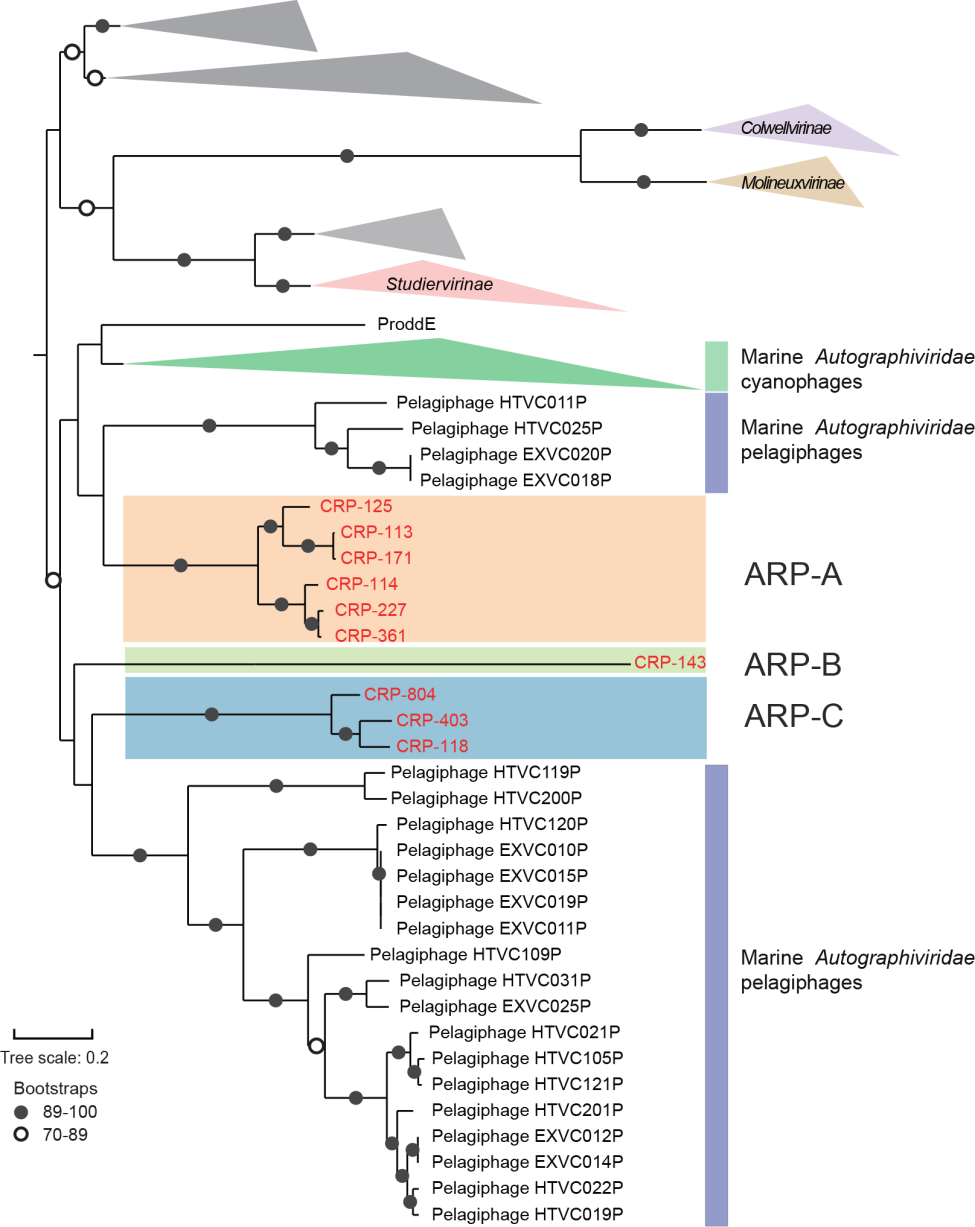
Phylogenomic analyses of *Autographiviridae* phages. A maximum-likelihood phylogenomic tree was reconstructed using concatenated sequences of ten conserved genes. The ten roseophages are shown in red. The roseophages were grouped into three groups (ARP-A, ARP-B and ARP-C) based on the phylogeny. Grey shading indicates *Autographiviridae* phages that have not been assigned to a known phage family.

Phylogenomic analysis was conducted based on concatenated conserved proteins to investigate the evolutionary relationships of *Autographiviridae* roseophages to other phages within the family *Autographivirinae*. The phylogeny showed that these new roseophages are phylogenetically more closely related to marine pelagiphages and cyanophages. At the same time, they are quite distinct from non-marine *Autographiviridae* phages (Fig. 3). The ten *Autographiviridae* roseophages are located in three distinct branches near pelagiphages, which are referred to as group ARP-A, ARP-B and ARP-C (Fig. 3). With respect to gene content, the ARP-B member CRP-143 has more variation (Fig. 1b). For example, integrase, endonuclease and exonuclease genes are present in all ARP-A and ARP-C genomes but absent in CRP-143. This suggests that CRP-143 may have an obligate lytic lifestyle and possess some differences in DNA metabolism.

**Fig. 4.**
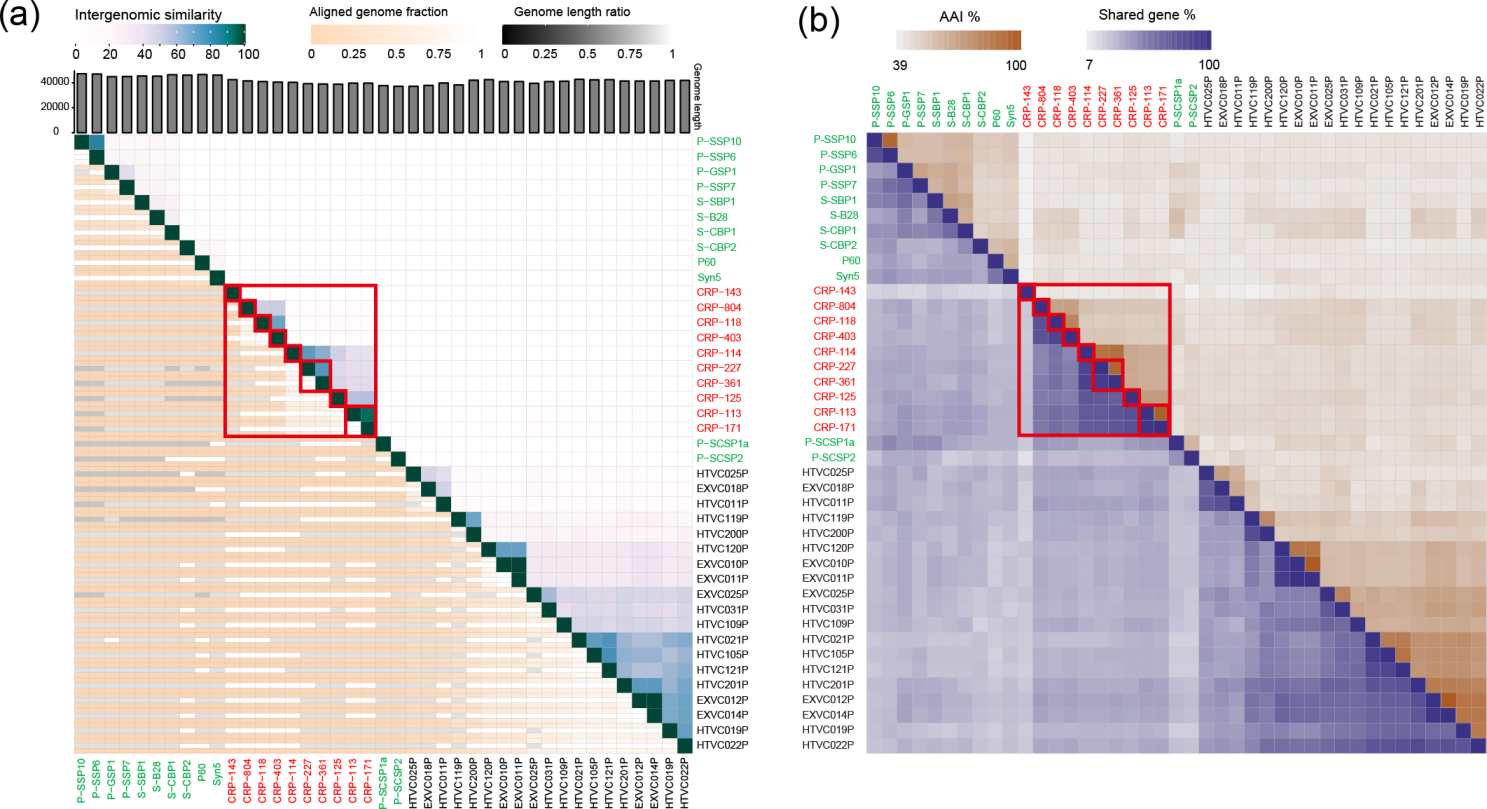
Heatmap showing (**a**) intergenomic similarities values and (**b**) shared gene percentages among *Autographiviridae* roseophages and other marine *Autographiviridae* phages. The *Autographiviridae* roseophages are shown in red. Roseophages classified in the same genus are in red boxes.

Furthermore, we used the virus intergenomic distance calculator (VIRIDIC) to calculate the intergenomic similarities between *Autographiviridae* phages. The result showed that *Autographiviridae* roseophages share low similarity with *Autographiviridae* cyanophages and pelagiphages (Fig. 4a). Average nucleotide identity (ANI) values calculated by VIRIDIC ranged from very low (<10 %) to high (94 %) among the ten roseophages, suggesting that they have high genome variation (Fig. 4a). The ten roseophages can be further classified into eight genera based on the proposed genus boundary cutoff of 70 % nucleotide identity (Fig. 4a). Furthermore, shared-gene analysis revealed that 19–90 % of genes are shared among the eight genera (Fig. 4b). ARP-B member CRP-143 is more distantly related to the other roseophages, sharing only 19–28 % of the genes with other *Autographiviridae* roseophages.

### Metabolic potential of marine *Autographiviridae* roseophages

Through functional annotation of the protein groups, we identified several AMGs from these roseophage genomes (Fig. 1). AMGs have been reported to enhance various host metabolic functions during phage infection to ensure the propagation of phage particles. The AMGs detected in these *Autographiviridae* roseophages include genes encoding glutaredoxin, nucleotide pyrophosphohydrolase and phosphate starvation-inducible.

All ten roseophages possess a glutaredoxin gene, a starvation-inducible protein gene (*phoH*) and a MazG-like pyrophosphatase gene (*mazG*). The phage-encoded *phoH* gene is frequently detected in various types of marine and non-marine phage genomes and is hypothesized to play an important role in assisting host phosphorous metabolism [71,73]. Glutaredoxin is a small redox protein that has been identified from many bacterial and phage genomes. *E. coli* phage T4 encoded glutaredoxin serves as an electron donor for T4 RNR, responsible for channelling the reducing potential of the cell into deoxynucleotide synthesis for phage DNA replication [74]. MazG protein is a regulator of nutrient stress and programmed cell death. The MazG of *E. coli* has been reported to counteract *mazEF* system-mediated cell death by decreasing the cellular level of (p)ppGpp [75]. Thus, it was hypothesized that phage-encoded MazG proteins play an important role in phage propagation by maintaining host cell survival and metabolism. Furthermore, a phage-encoded MazG was reported to be able to deplete ppGpp and prevent hosts from cell suicide caused by abortive infection (Abi), ensuring successful phage propagation [76]. In contrast, a study of a cyanophage S-PM2 encoded MazG revealed that this MazG has no binding or hydrolysis activity against (p)ppGpp but can hydrolyse dGTP and dCTP, and thus was speculated to play a role in hydrolysing high G+C host genomes for phage replication [77]. The functions of these roseophage-encoded MazG proteins remain unknown and warrant further investigation.

### Distribution of *Autographiviridae* roseophages in global oceans

**Fig. 5.**
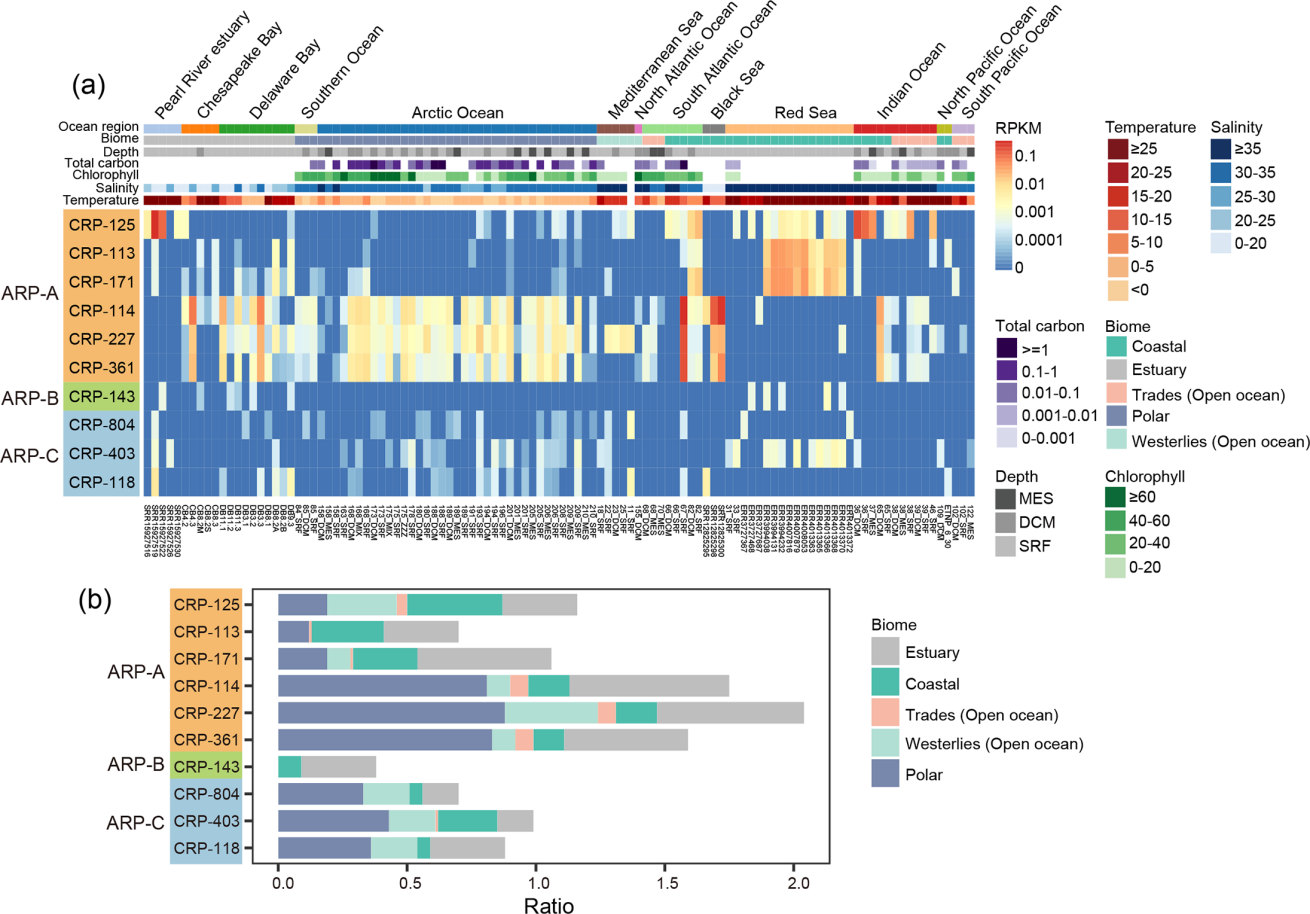
Biogeographical distribution of marine *Autographiviridae* roseophages across the global ocean. (**a**) Heat map of RPKM values of *Autographiviridae* roseophages in different marine viromes. Only those stations where *Autographiviridae* roseophages were present are shown. (**b**) The proportion of stations at which each *Autographiviridae* roseophage can be detected.

We analysed 220 marine viromic datasets covering a wide range of global ocean regions from pole to pole and different water layers of five marine biomes to elucidate and compare the distribution and relative abundance of the *Autographiviridae* roseophages (Table S2). About half of the marine viromic datasets were found to contain *Autographiviridae* roseophages. Overall, these *Autographiviridae* roseophages can be detected in various marine viromes, predominantly occurring in estuarine, polar and coastal stations (Fig. 5). They were frequently detected in the temperate Chesapeake Bay and Delaware Bay stations (Fig. 5a). Some roseophages were also found in the subtropical Pearl River Estuary (Fig. 5a). Six of the ten roseophages can be detected in over one-third of the polar stations (Fig. 5b). Some roseophages were also widely distributed in coastal stations. The distribution of *Autographiviridae* roseophages mirrors the distribution patterns of their *Roseobacter* hosts. Of the ten roseophages, eight infect the RCA strains. The *Roseobacter* RCA lineage is the most dominant lineage within the *Roseobacter* group and is prevalent in temperate to polar oceans [78,80]. Another roseophage infects the *Roseobacter* CHUG strain, which is also widely spread in global oceans [65].

Vertically, these *Autographiviridae* roseophages also exhibit distribution characteristics similar to roseobacters [30]. They were mainly detected in surface (SRF) and deep chlorophyll maxima (DCM) zones and occasionally detected in mesopelagic (MES) waters (Figs 5a and S3a). Most roseophages have a higher relative abundance in the SRF and DCM waters compared to the MES waters (Fig. S3a).

Of these ten roseophages, CRP-227, CRP-114 and CRP-361 in group ARP-A exhibit a broader distribution profile (Fig. 5). These three roseophages infect *Roseobacter* RCA strains. They were present in >80 % of the polar stations and about half of the estuarine stations, and can also be detected in some coastal, trade and westerlies stations. In addition, they had significantly higher relative abundance compared to other roseophages in polar stations (Figs 5 and S3b). This suggests that their hosts may have a wider salinity and temperature adaptation. In contrast, ARP-B member CRP-143, also infecting the RCA strain, exhibited the most limited distribution, being detected only in estuarine and coastal stations (Fig. 5). The distribution of phages is generally related to the distribution of their hosts. However, we found that some roseophages infecting the same hosts differed in their distribution pattern. This may be due to their differences in genomic and physiological characteristics.

A linear regression analysis was performed to reveal the correlations between environmental factors and the relative abundance of each roseophage. The results revealed that the relative abundance of five roseophages (CRP-114, CRP-227, CRP-361, CRP-804 and CRP-118) was positively correlated with total carbon (Fig. S4). It has been reported that relative abundances of their hosts are positively correlated with total carbon [65]. In contrast, the relative abundance of the other five roseophages (CRP-125, CRP-113, CRP-171, CRP-143 and CRP-403) did not show this correlation, but their relative abundance was positively correlated with temperature (Fig. S4). This suggests that these roseophages have limited adaptation to low temperature, although the underlying mechanism remains unclear. Regarding other environmental parameters, some roseophages showed a positive correlation with ammonium, nitrate or nitrite. The relative abundance of all ten roseophages was not correlated with chlorophyll a (Chl-a), depth or phosphate. We found that some phages from the same group showed different correlations with environmental factors (Fig. S4), suggesting their different adaptations to host and environmental factors.

### Conclusions

Continuous laboratory efforts have been made to culture new marine phages and investigate their evolution and ecological function. In this study, we isolated and reported genomes of the first *Autographiviridae* phages infecting marine *Roseobacter* strains, revealing their similarity to other *Autographiviridae* phages and unique characteristics. These roseophages represent three novel groups in the family *Autographiviridae* and were widely distributed in global oceans. Our work deepens knowledge of the diversity and evolution of *Autographiviridae* phages, highlighting the ecological potential of *Autographiviridae* phages by infecting diverse hosts. Based on these systems, further comprehensive analysis on the genetic diversity of marine *Autographiviridae* phages and their interactions with bacterial hosts could be performed in the future.

## supplementary material

10.1099/mgen.0.001240 Fig. S1.

10.1099/mgen.0.001240 Table S1.

## Abbreviations

AAI: average amino acid identity
AMG: auxiliary metabolic gene
ANI: average nucleotide identity
DCM: deep chlorophyll maximum
DNAP: DNA polymerase
MES: mesopelagic
RNAP: RNA polymerase
RNR: ribonucleotide reductase
RPKM: reads per kilobase per million reads
SRF: surface
SSB: single-stranded DNA binding protein
TEM: transmission electron microscopy
TerL: terminase large subunit
