# Supplementary material for: Genome sequences of the first Autographiviridae phages infecting marine Roseobacter
Source: Microb Genom. 2024 Apr 17;10(4):001240. doi: 10.1099/mgen.0.001240 (PMC11092137; doi:10.1099/mgen.0.001240)
Supplement: Fig. S1. [file mgen-10-01240-s001.pdf]

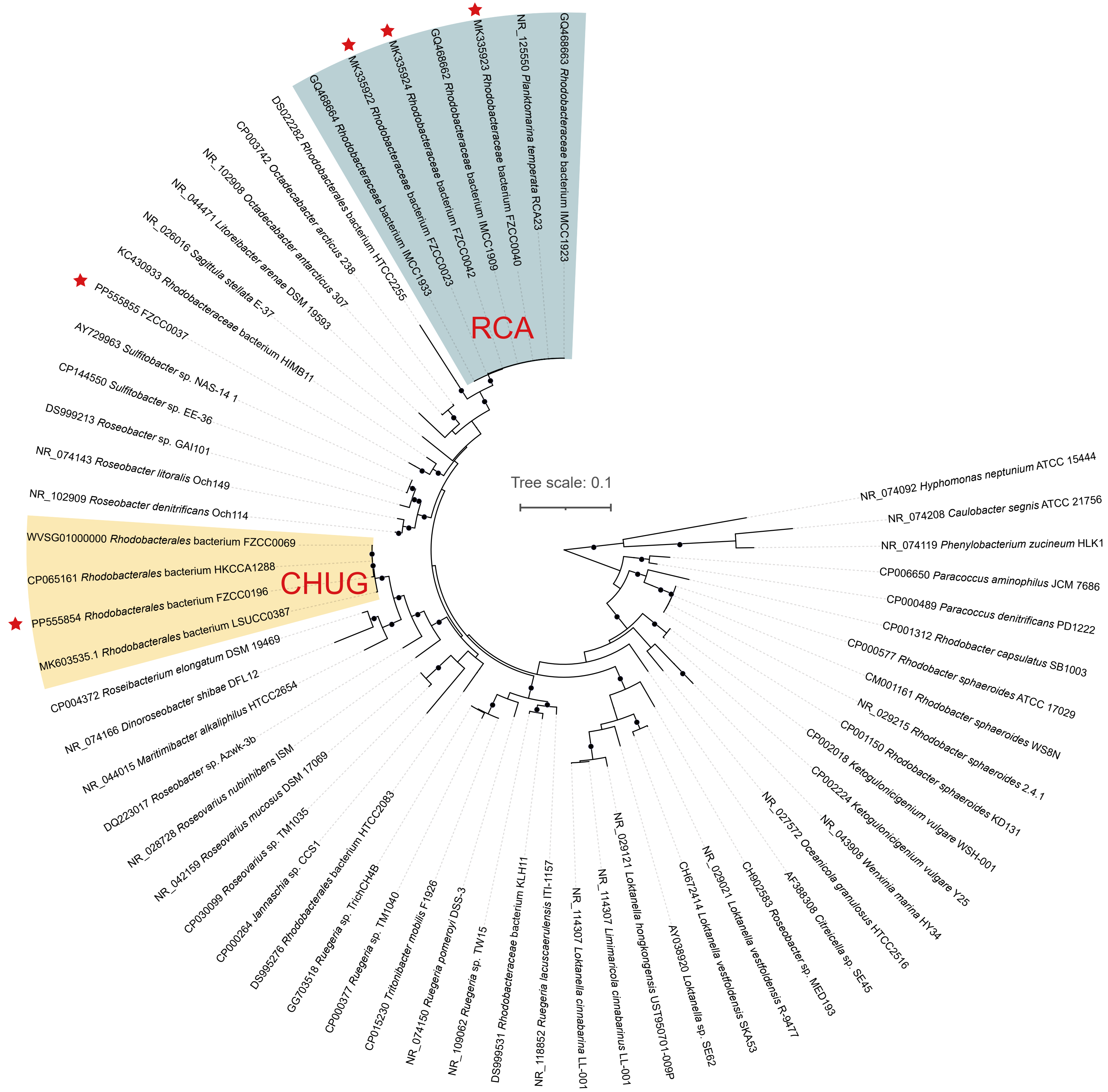

Supplementary Figure S1 Phylogenetic tree based on the 16S rRNA gene sequences of Roseobacter

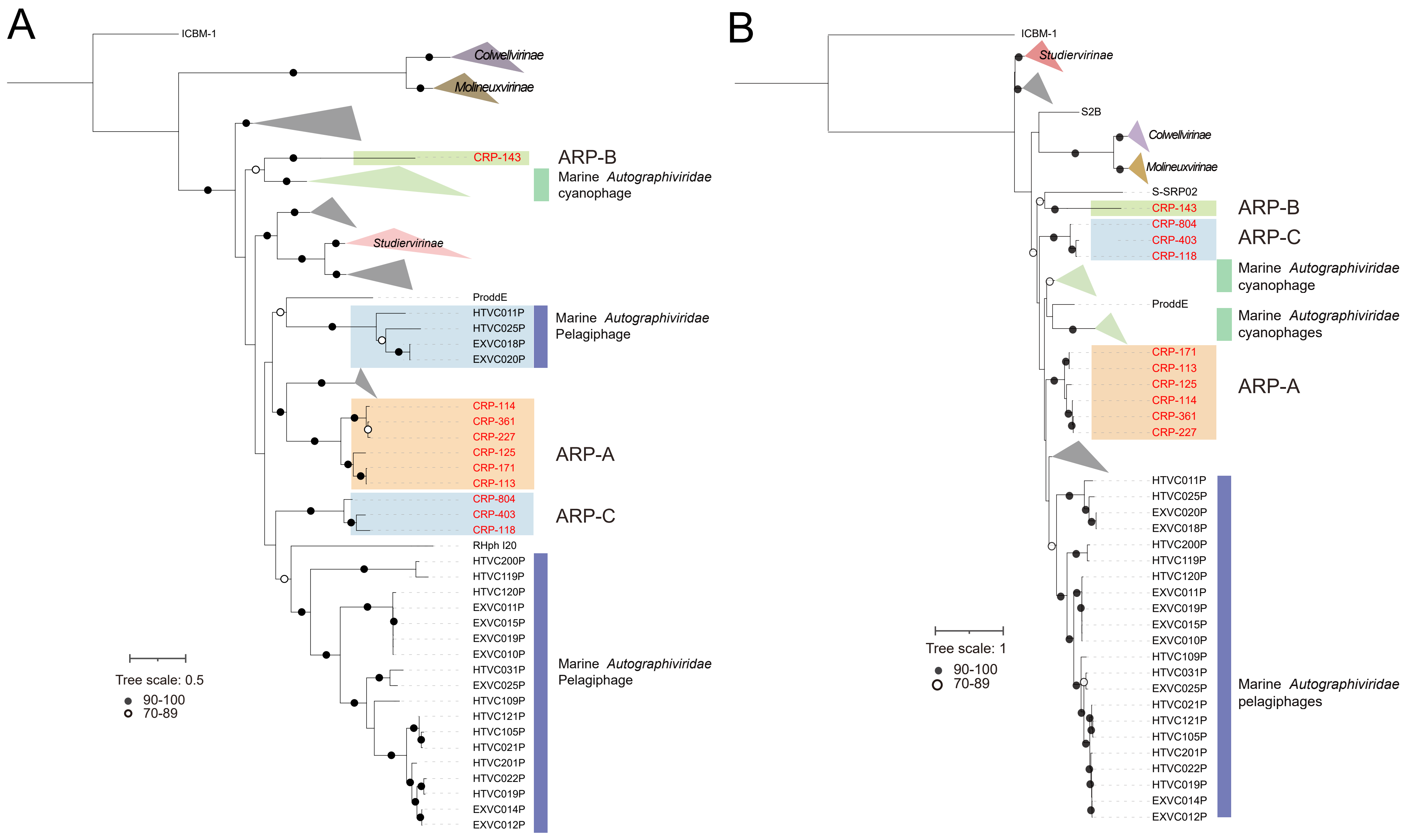

Supplementary Figure S2 Unrooted maximum-likelihood phylogenetic tree of *Autographiviridae* phages DNAP (A) and TerL (B). The roseophages isolated in this study are shown in red.

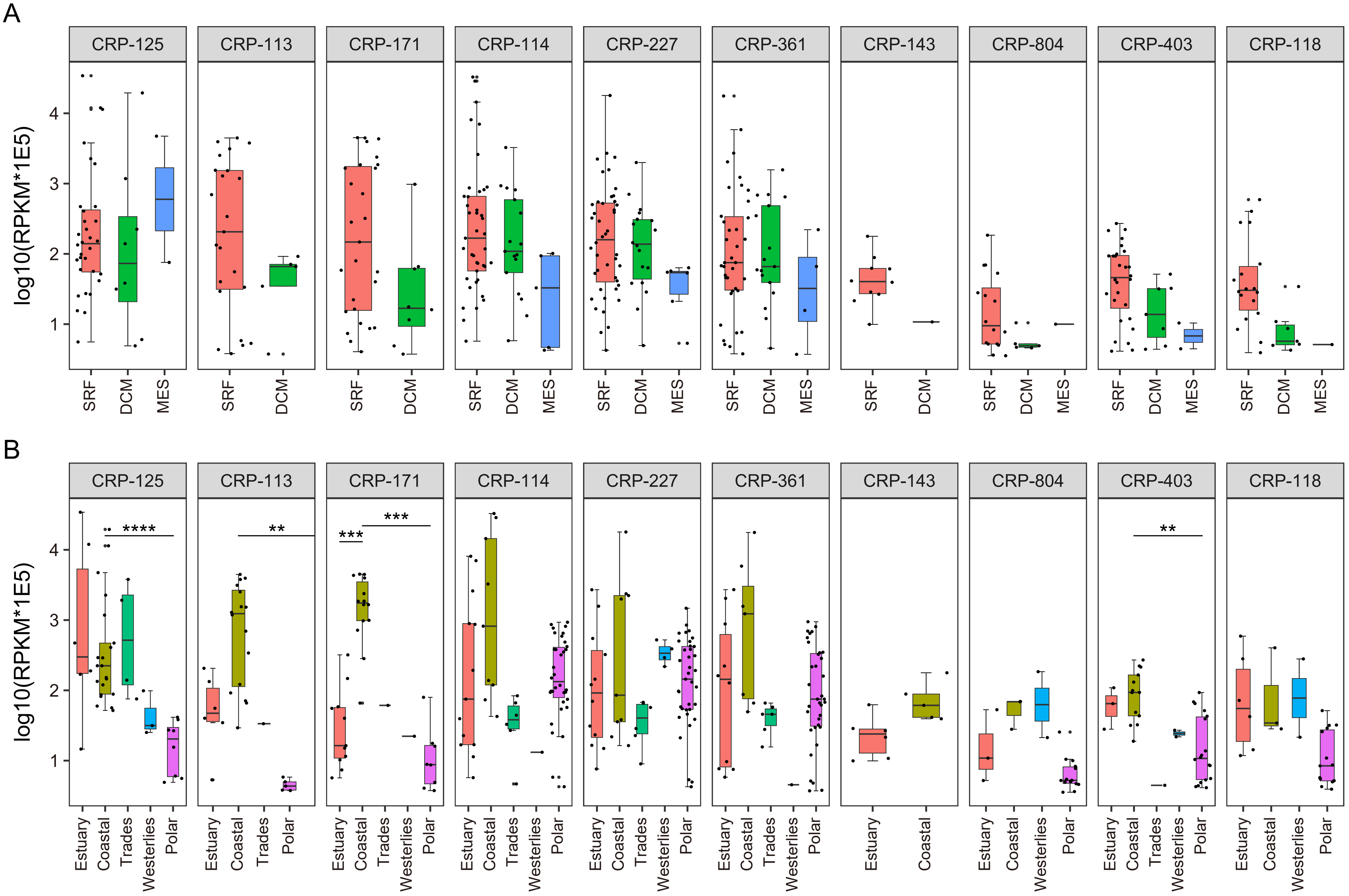

Supplementary Figure S3 The relative abundance of marine *Autographiviridae* roseophages in different marine environments. (A) Box plot showing the relative abundance of each *Autographiviridae* roseophage in different oceanic layers. (B) Box plot showing the relative abundance of each *Autographiviridae* roseophage in different marine biomes. The significance of pairwise comparisons calculated using the two-tailed Mann–Whitney U test was shown, with the asterisk corresponding to the pvalue (* pvalue < 0.05, **pvalue < 0.01, ***pvalue < 0.001).

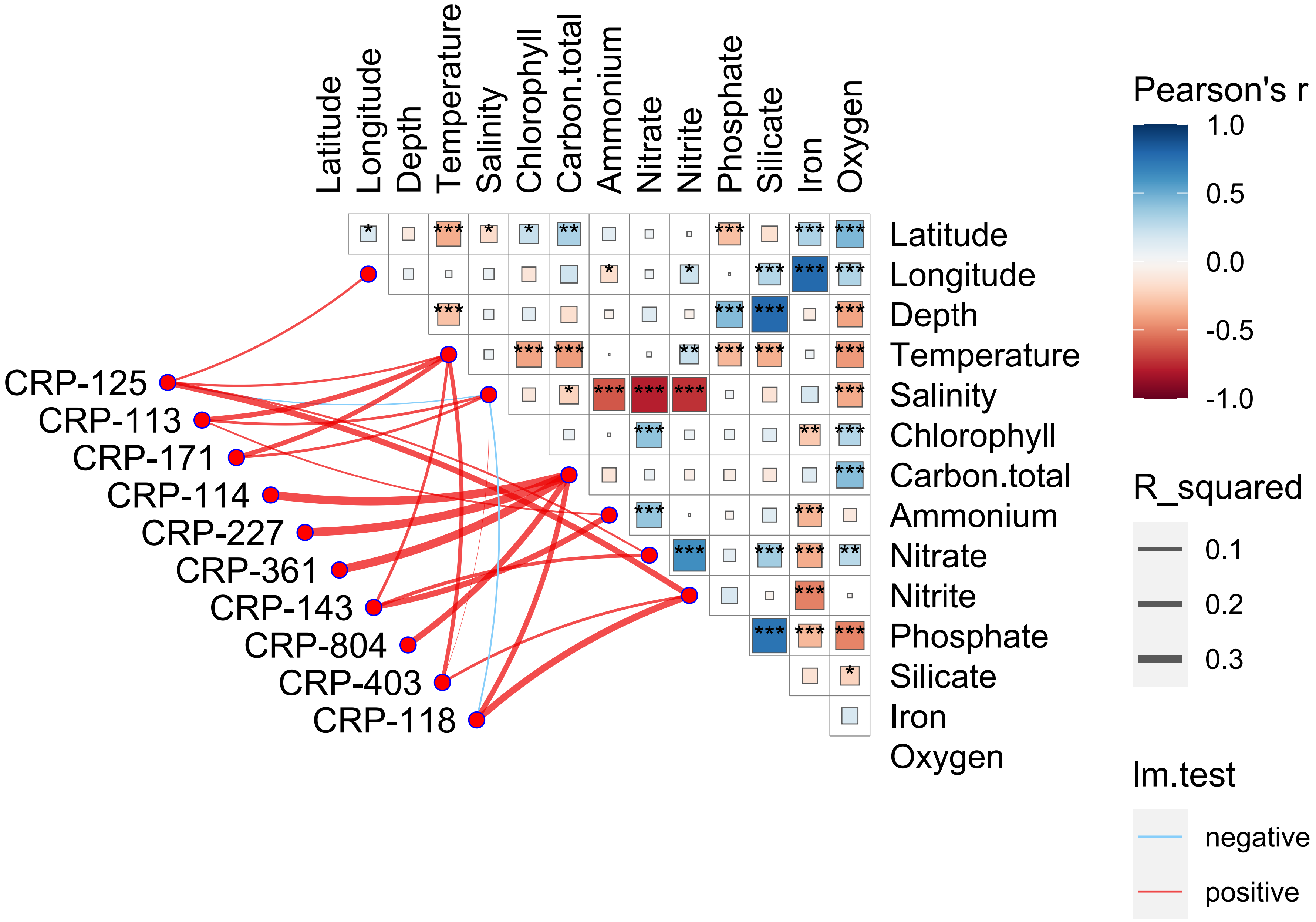

Supplementary Figure S4 Environmental drivers of marine *Autographiviridae* roseophages. The correlation of phage distribution with environmental factors was calculated using linear regression. The line width corresponds to the R-square coefficient of the linear regression. The red and blue lines represent statistically significant positive and negative correlations (p <0.05) Pearson correlation coefficients between pairwise comparisons of environmental factors are represented with gradient colors. The Pearson's correlation significance of pairwise comparisons was shown with the asterisk corresponding to the pvalue (* pvalue < 0.05, **pvalue < 0.01, ***pvalue < 0.001).
